# Early Administration of Protein in Critically Ill Patients: A Retrospective Cohort Study

**DOI:** 10.3390/nu11010106

**Published:** 2019-01-07

**Authors:** Itai Bendavid, Oren Zusman, Ilya Kagan, Miriam Theilla, Jonathan Cohen, Pierre Singer

**Affiliations:** 1 Department of General Intensive Care and Institute for Nutrition Research, Rabin Medical Center, Beilinson Hospital, 49100 Petah Tikva, Israel; ilyak@clalit.org.il (I.K.); miriamt@clalit.org.il (M.T.); jdcspc@gmail.com (J.C.); psinger@clalit.org.il (P.S.); 2 Sackler School of Medicine, Tel Aviv University, 39040 Tel Aviv, Israel; orenzusman@gmail.com; 3 Department of Cardiology, Rabin Medical Center, Beilinson Hospital, 49100 Petah Tikva, Israel; 4 Nursing Department, Steyer School of Health Professions, Sackler School of Medicine, Tel Aviv University, 39040 Tel Aviv, Israel

**Keywords:** critically ill, nutrition, protein, early, mortality

## Abstract

It is currently uncertain whether early administration of protein improves patient outcomes. We examined mortality rates of critically ill patients receiving early compared to late protein administration. This was a retrospective cohort study of mixed ICU patients receiving enteral or parenteral nutritional support. Patients receiving >0.7 g/kg/d protein within the first 3 days were considered the early protein group and those receiving less were considered the late protein group. The latter were subdivided into late-low group (LL) who received a low protein intake (<0.7 g/kg/d) throughout their stay and the late-high group (LH) who received higher doses (>0.7 g/kg/d) of protein following their first 3 days of admission. The outcome measure was all-cause mortality 60 days after admission. Of the 2253 patients included in the study, 371 (36%) in the early group, and 517 (43%) in the late-high group had died (*p* < 0.001 for difference). In multivariable Cox regression analysis, while controlling for confounders, early protein administration was associated with increased survival (HR 0.83, 95% CI 0.71–0.97, *p* = 0.017). Administration of protein early in the course of critical illness appears to be associated with improved survival in a mixed ICU population, even after adjusting for confounding variables.

## 1. Introduction

Critically ill patients experience metabolic changes that may carry deleterious effects [1], including protein catabolism and skeletal muscle wasting [2], which may later translate into increased morbidity and mortality [3]. Survivors may suffer from low muscle mass and prolonged weakness [4]. Adequate and prompt protein provision to critically ill patients has been recognized as a cornerstone in the care of the critically ill, mainly based on an earlier prospective randomized controlled trial [5] and on more recent observational studies [6,7,8,9,10,11]. These have resulted in the current guidelines [12], which endorse higher protein targets than previously recommended, generally above 1.2 g/kg/d. However, limited data from randomized controlled studies make this recommendation weak in regards to what dose of protein to administer. Some studies have shown a beneficial effect even in doses higher than that commonly recommended, namely 1.2–1.5 g/kg/d [9,13,14]. Some studies [15,16], albeit not all [17], have shown benefit in the setting of acute kidney injury, leading some experts to advocate 2–2.5 g/kg/d in certain patient populations [18]. However, this beneficial effect was not found in a recent randomized controlled trial [19], resulting in ongoing uncertainty. A second key question when prescribing nutrition relates to the optimal timing for protein delivery in critically ill patients. Existing data are inconclusive and in fact current guidelines [12,20] do not specifically address this issue. In a post hoc analysis, Casaer et al. suggested that early administration (day 3) of protein was harmful to ICU patients in terms of mortality [21]. While it has been suggested to use indirect calorimetry for the accurate evaluation of energy needs when provided by early enteral nutrition if feasible, it remains uncertain whether and for whom trophic or hypocaloric targets should be preferred [22]. In this regard, adequate protein provision is harder to achieve when lower energy needs are targeted. A call for further research regarding optimal timing for the delivery of protein has been issued [23]. In an effort to increase the current body of evidence, we conducted a retrospective study of critically ill patients, specifically examining outcome in relation to timing of protein delivery.

## 2. Materials and Methods

### 2.1. Patients

We included all patients who were hospitalized from 2003–2015 in a 16-bed, mixed medical-surgical ICU at a tertiary-care, university affiliated hospital. Data were drawn from a computerized patient record system (MetaVision ICU^®^, iMDSoft, Tel Aviv, Israel). For the statistical analysis, we included only patients with an ICU stay of > 96 h of evaluable nutrition days in order to reduce any possible bias caused by short stay, early mortality and the expectation that the effect of nutrition might necessitate at least this duration of exposure [24]. The count of length of stay and evaluable nutritional days started at the hour of arrival in the ICU.

### 2.2. Study Design

Retrospective cohort. Demographic data collected included age, sex, height and weight, admission SOFA score, admission category (medical, surgical or trauma), and admission diagnosis (cardiovascular, respiratory, and sepsis which were not mutually exclusive). Nutritional parameters noted included route of feeding (enteral, parenteral, or both), insulin therapy (units/day), and the amount of total calories and protein administered daily until ICU death, discharge from the ICU, or the start of exclusive oral feeding. The mean amount of gram protein per kg per day was calculated for the first three days; the relation of protein was analyzed both as a continuous and categorical variable. Observational studies regarding protein administration have shown low utilization of protein in patients receiving 60% of that prescribed, which equates to around 0.7 g/kg/d (or specifically to 0.5 g/kg/d in that study) [8]. Others [25,26] have used 0.8 g/kg/d. In earlier studies from our center [9], we estimated that 0.7 g/kg/d would be close to the mean and median values of actual delivered protein. We therefore divided our patient cohort into 2 groups, namely those who failed to receive >0.7 g/kg/d in the first 3 days, who constituted the “late” group, and those who received >0.7 g/kg/d, who constituted the “early” group. The late group was further subdivided into a late-low group (LL), who received low protein intake (<0.7 g/kg/d) throughout their stay, and the late-high group (LH), who received higher amounts (>0.7 g/kg/d) of protein. Non-nutritional calories administered in the form of glucose infusions and propofol were included as administered calories. Patients who were readmitted to the ICU were not included. 

### 2.3. Outcome

The outcome measure was all-cause mortality at 60 days from admission. This included mortality during and after ICU stay. In order to mitigate the possible effect of the duration of exposure to nutrition on the results, we planned on adjusting for total evaluable nutrition days, and year of hospitalization, as the cohort spanned 13 years. In addition, in order to better adjust for weight and SOFA differences, we used propensity score adjustment in another analysis. Since death date is updated in our computer records by the Ministry of Health, we were able to record both in-hospital and post-hospital discharge death. 

### 2.4. Ethics

The study was approved by the Rabin Medical Center institutional review board who waived the requirement for consent.

### 2.5. Statistical Analysis

Continuous normally distributed variables are presented as means ± standard deviations (SD) and compared using the Student’s t-test. Ordinal or non-normally distributed variables are presented by median and interquartile range (IQR) and compared using the Wilcoxon rank sum test. Normality was assessed using the Shapiro-Wilk test. Categorical variables are compared using the chi square test.

An adjusted Cox multivariable model, with covariates selected based on univariate analysis, was fitted. Multicollinearity was assessed by examining variation inflation factors and *R*^2^. For the sensitivity analysis, we used multinomial propensity scores adjustment serving as weights. All statistical procedures were carried out in R (Vienna, Austria, 2017).

## 3. Results

**Figure 1:**
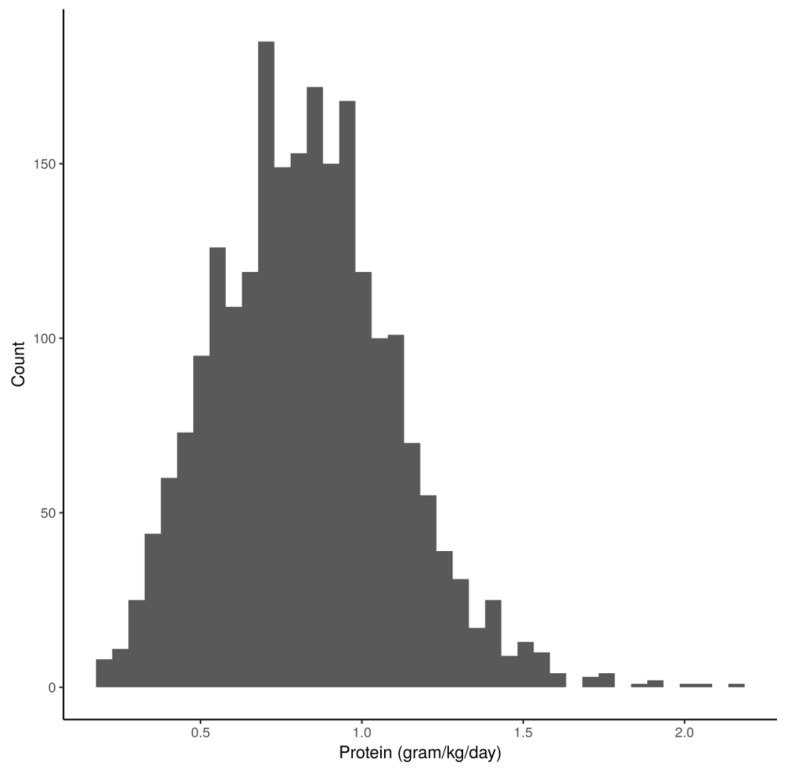
Histogram of the number of patients according to daily administered protein adjusted for weight. Legend: On the X axis is the amount of protein administered daily per Kg, on the Y axis is the number of patients receiving that mean amount.

A total of 2253 patients were included in the study. Mean protein delivery for the overall cohort was 0.64 (±0.33) g/kg for the first 3 days, and 0.83 (±0.28) g/kg throughout the hospitalization period. A histogram of administered protein values is presented in Figure 1. 

When examined as a continuous variable, the amount of protein administered in the first 3 days adjusted for other protein sources, weight, age, total calories received, SOFA score, and parenteral nutrition, was associated with a decreased mortality (HR 0.9, 95% CI 0.82–0.99, *p* = 0.03). A total of 1040 patients were included in the early protein (EP) group and 1213 in the late (LP) group. Patient characteristics are shown in Table 1. 

**Figure 2:**
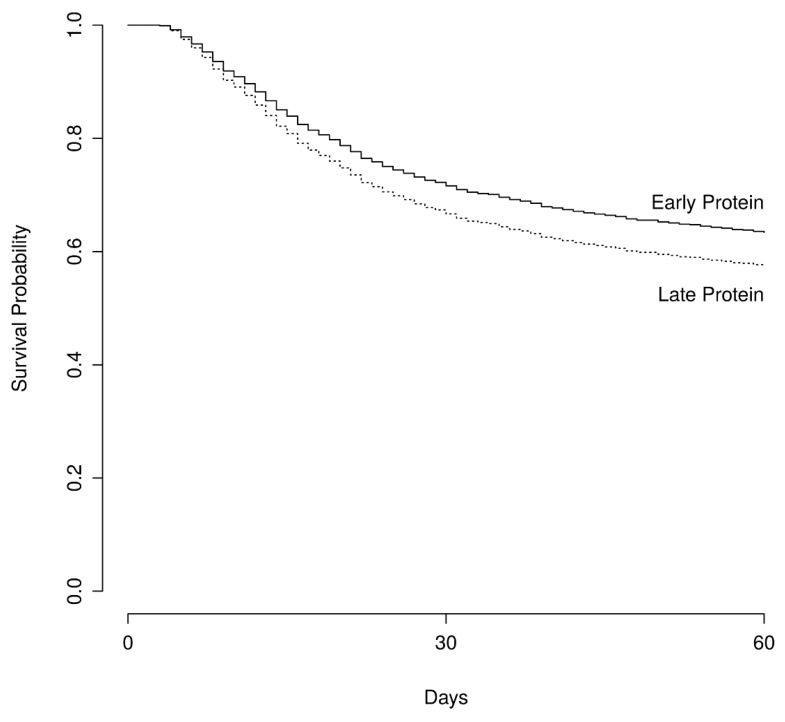
Survival. This figure shows adjusted survival estimates of the survival curves among patients receiving early or late protein. The Hazard Ratio for death was calculated at 0.83 (95%, CI 0.71–0.97) with a *p* value of 0.01 using a multivariable Cox regression analysis that controlled for age, sex, weight, parenteral nutrition, mean delivered calories, mean daily protein received after the first 3 days, administration of vasopressors, SOFA score, and total hospital stay.

Patients in the EP group had a lower BMI and lower admission SOFA score, but were of similar age and category of hospitalization. At 60 days post admission, 371 (36%) in the EP and 517 (43%) in the LP group had died (*p* < 0.001 for difference), as shown in Figure 2. 

In multivariable Cox regression analysis, increased survival was demonstrated in the EP group (HR 0.83, 95% CI 0.71–0.97, *p* = 0.017), while controlling for age, sex, weight, parenteral nutrition, mean delivered calories, mean daily protein received after the first 3 days, administration of vasopressors, SOFA score, year of study, and total hospital stay (Supplemental Digital Content—Table S1).

### 3.1. Comparison with Late-High and Late-Low Protein

Of the 1213 patients in the late protein group, 488 received low protein (<0.7 g/kg/d) throughout their stay (late-low group, LL), while 725 patients received higher (>0.7 g/kg/d) protein following their first 3 days of admission (late-high group, LH). Comparison of patient characteristics in the three groups is presented in Table 2.

**Figure 3:**
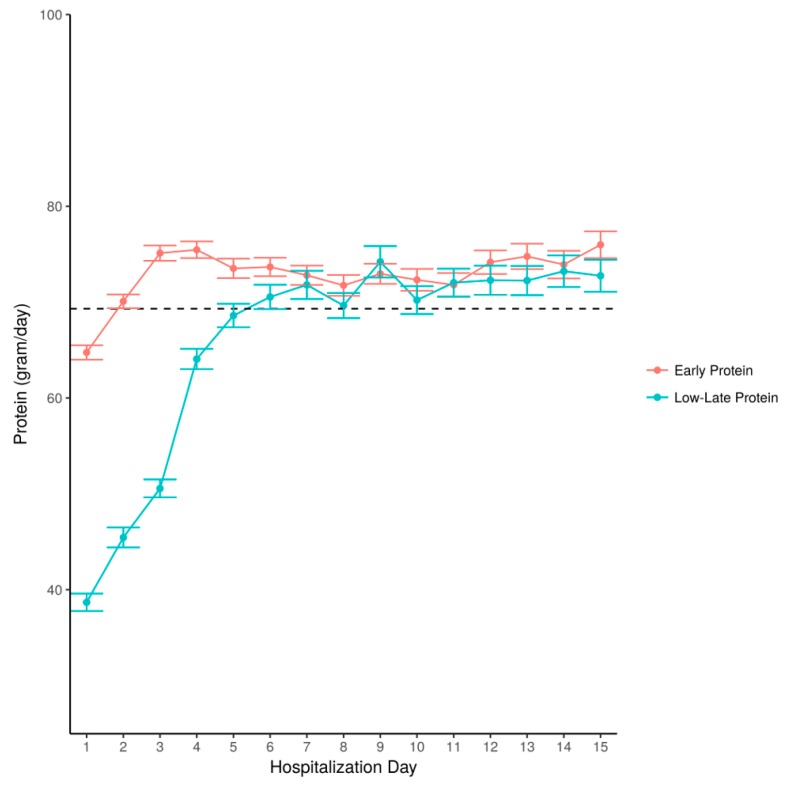
Amount of protein administered by hospitalization day—a comparison between the early protein and the late-low groups. Legend: The amount of daily protein, presented as grams per day, is shown according to hospitalization day. Stratification according to patient groups: those who received early protein and those who received late-low protein. Dotted line—mean amount for all patients throughout hospitalization.

After the first three days, the EP group still received higher amounts than the LH group, although the difference was smaller (1.01 vs 0.93 g/kg/d, *p* < 0.001. Patients in the LL group achieved the mean amount of protein for patients throughout hospitalization (Figure 3).

After 60 days, 222 (45%) of the LL group, and 295 (41%) of the LH group had died, both significantly more than the EP group (*p* < 0.001). Compared to the EP group, in multivariate analysis, after controlling for age, sex, weight, parenteral nutrition, mean delivered calories, mean daily protein received after the first 3 days, administration of vasopressors, SOFA score, and total hospital stay, a significant association with mortality was found for the LH group (HR 1.21, 95% CI 1.03–1.42, *p* = 0.02) while there was a trend towards increased mortality in the LL group (HR 1.24, 95% CI 0.97–1.57, *p* = 0.08).

### 3.2. Sensitivity Analysis

In order to take major confounders further into account, we used multinomial propensity score adjustment using BMI and SOFA score and added regression adjustment for delivered calories in the first 3 days relative to the estimated requirement. This was in addition to controlling for the other variables described. Compared to the group with adequate administration of protein (the EP group), the groups who received late and low administration of protein (LL group) had higher associated mortality (HR 1.28, 95% CI 1.01–1.63, *p* = 0.046). No significant difference in associated mortality was found between the LL and late-high (LH) group (LL for HR 1.11, 95% CI 0.88–1.39, *p* = 0.36).

## 4. Discussion

The question regarding the optimum amount and timing of protein administration for critically ill patients remains complex. In a randomized multicenter trial [27] comparing early with late administration of parenteral nutrition, Casaer et al. found early parenteral nutrition to be associated with more frequent infections, longer periods of ventilation and renal replacement therapy, increased cholestasis, and higher hospital costs. In a later post-hoc analysis [23] of specific macronutrients and delivery routes, early protein delivery, irrespective of the route, also appeared to be detrimental. In a systematic review and meta-analysis of published data until 2015 [28], protein delivery in high doses (1.02 g/kg/d) was not associated with increased survival rates compared to low doses (0.67 g/kg/d). In a recent prospective randomized trial [19] of 200 patients, goal-directed nutritional therapy was compared to standard of care and no significant difference in outcome was found. However, in 2014 Weijs et al. [25] published a prospective study of 843 mixed critically ill patients and found early administration of protein improved survival in these patients. This effect on mortality, however, did not extend to 117 septic patients. In an observational study, Song et al. [29] found improved survival in patients achieving over 90% of their protein target within the first week, regardless of whether energy targets were met or not. In a large prospective, single-blind study [30], no effect on mortality was noted when patients received more energy and protein via the parenteral route; however, improved weaning from mechanical ventilation and less muscle mass loss was demonstrated. A further two retrospective studies also found that failure to provide adequate protein was associated with worse outcomes [26,31], although in the first of these early high protein intake (within 3–5 days) was associated with worse outcomes as well. Apart from sepsis, a key variable might be renal function, as patients with normal function were shown to benefit more [32]. The association with better survival appears to possibly contradict the EAT-ICU [19] study that showed no effect on 90-day mortality. Singer et al. [33] explained this finding by the large proportion of septic patients, who might benefit less from high protein administration [18]. In addition, despite the robust methodology, the sample size was modest, possibly resulting in missing a smaller effect of interventions. Thus, a HR of 1.3, close to the association found in this study, could only have been detected with the inclusion of roughly 1100 patients (with a power of 0.8 and alpha of 0.05). This might partly explain why an association was found in larger retrospective trials but not in a RCT. It is remarkable to note that even patients with sepsis and multi-organ failure have a preserved ability to digest and absorb protein. This was demonstrated by Beale et al. by measuring the plasma levels of serine, ornithine, arginine and glycine [34].

The conflicting studies mentioned above leaves the matter of protein provision at a problematic state [35], since practicing physicians might equate “absence of evidence” with “evidence of absence”, and possibly neglect energy or protein provision under the false impression that it is not important. It also appears that it is difficult to achieve the recommended 1.2–1.5 g/kg/d of protein in real-life practice. The “optimal” amount of protein remains elusive, but we have shown that even using a cutoff of 0.7 g/kg/d can be beneficial. The use of specialized protein-rich formulas may enable the provision of higher protein doses without the risk of overfeeding during the early ICU admission period [36]^.^ It remains to be seen how sepsis and kidney function affect protein requirements, and what exact amount of protein is needed. The recent ESPEN guidelines [37] recommend the progressive administration of 1.3 g/kg/day; our study explored how this progression could be performed.

In our study, the increased administration of protein in the first 3 days was associated with better 60-day survival. This association was observed as a continuous or dichotomous variable and remained evident after adjusting for demographic and anthropomorphic variables, for markers of physiology and disease severity, for confounders that may affect nutrition delivery and utilization, and after the use of propensity scores for BMI and SOFA score. The mean protein delivered was 50 g/d or 0.64 g/kg/d, which is much less than the recommended 1.2 g/kg/d.

The strength of this study lies in the relatively large number of participants, which allows both to better control for confounders and to identify smaller associations.

The study has several limitations. First, by its retrospective nature it demonstrates association and not causation, and the results might be biased. Specifically in nutrition studies, there may be a concern that more “difficult”’ patients or patients that died early received less protein, and protein provision itself is confounded by weight and general nutritional provision. Second, we used specific cutoffs which might be perceived as low (0.7 g/kg/d). Finally, we examined only the association with mortality. We attempted to address these limitations in the following manner. The cohort of patients with a minimum length of stay was restricted, an attempt was made to control for multiple variables, and differences according to protein use after 3 days including a “late-high” group (those who received protein in amounts similar to those in the early protein group after 3 days). Regarding a specific protein cut-off level, some studies have used 0.8 g/kg/d [25,26], while we demonstrated that 0.6–0.7 g/kg/d was the mean delivered amount [8,9]. We have thus chosen a practical, common threshold that serves for comparison purposes, rather than as a target. Randomized controlled studies should be encouraged, but the large sample sizes required make these difficult to conduct so that in the interim reliance is placed on observational data.

## 5. Conclusions

Most patients do not achieve guideline recommended targets for protein, especially in the first days following admission. In our study, patients receiving > 0.7 g/kg/d of protein in the first 3 days of ICU hospitalization had higher 60-day survival compared to those who received less. These results should be examined in a large randomized trial. Further research is necessary in order to better stratify those who may benefit from earlier or higher protein intakes.

## Figures and Tables

**Table 1:** Patient Characteristics—Comparison between Early and Late Protein.

Characteristic	Overall	Early Protein	Late Protein	*p*-Value
*n*	2253	1040	1213	
Age	58.96 (18.32)	59.22 (19.18)	58.74 (17.55)	0.537
Male Sex	1447 (64.2)	651 (62.6)	796 (65.6)	0.147
Weight	80.22 (19.40)	73.74 (13.91)	85.78 (21.60)	<0.001
Height	1.70 (0.09)	1.69 (0.09)	1.70 (0.09)	<0.001
Body surface area	1.90 (0.22)	1.84 (0.18)	1.96 (0.23)	<0.001
Body mass index	27.91 (7.09)	25.80 (4.47)	29.72 (8.32)	<0.001
SOFA score	8.03 (3.57)	7.69 (3.30)	8.32 (3.77)	<0.001
Ventilated	2218 (98.4)	1031 (99.1)	1187 (97.9)	0.023
Vasopressors	884 (39.2)	396 (38.1)	488 (40.2)	0.317
Cardiac	365 (16.2)	171 (16.4)	194 (16.0)	0.817
Sepsis	387 (17.2)	169 (16.2)	218 (18.0)	0.306
Surgical	656 (29.1)	311 (29.9)	345 (28.4)	0.475
Trauma	252 (11.2)	131 (12.6)	121 (10.0)	0.057
Respiratory	379 (16.8)	177 (17.0)	202 (16.7)	0.861
Enteral Calories	1334.64 (650.13)	1457.64 (545.94)	1227.77 (711.85)	<0.001
Parenteral Calories	346.28 (581.76)	317.51 (578.83)	371.29 (583.39)	0.034
Total Calories	1684.17 (646.56)	1780.17 (444.29)	1601.87 (769.97)	<0.001
Calculated energy expenditure	2005.55 (485.01)	1843.56 (347.63)	2144.44 (540.05)	<0.001
Administered calories to energy expenditure	0.88 (0.37)	0.98 (0.25)	0.78 (0.42)	<0.001
Significant parenteral nutrition	311 (13.8)	127 (12.2)	184 (15.2)	0.049
Total protein delivered	1058.74 (929.18)	1193.92 (939.76)	942.85 (904.45)	<0.001
Daily protein delivered	63.59 (19.00)	71.18 (16.79)	57.08 (18.35)	<0.001
Daily protein, g/kg	0.83 (0.28)	0.98 (0.22)	0.69 (0.26)	<0.001
Daily protein, g/kg, first 3 days	0.64 (0.33)	0.93 (0.19)	0.39 (0.19)	<0.001
Daily protein, g/kg, other days	0.87 (0.30)	1.01 (0.24)	0.75 (0.30)	<0.001

For continuous variables data presented as mean (sd) and discrete variables as *n* (%). SOFA: Sequential Organ Failure Assessment.

**Table 2:** Patient Characteristics—Comparison between Early, Late-Low, and Late-High Protein.

Characteristic	Early Protein	Late-Low Protein	Late-High Protein	*p*
*n*	1040	488	725	
Age	59.22 (19.18)	60.39 (16.44)	57.64 (18.18)	0.031
Male Sex	651 (62.6)	319 (65.4)	477 (65.8)	0.324
Weight	73.74 (13.91)	96.81 (24.16)	78.35 (15.86)	<0.001
Height	1.69 (0.09)	1.71 (0.09)	1.70 (0.09)	<0.001
Body surface area	1.84 (0.18)	2.07 (0.23)	1.89 (0.19)	<0.001
Body mass index	25.80 (4.47)	33.38 (10.09)	27.25 (5.66)	<0.001
SOFA score	7.69 (3.30)	8.43 (3.82)	8.25 (3.74)	<0.001
Ventilated	1031 (99.1)	470 (96.3)	717 (98.9)	<0.001
Vasopressors	396 (38.1)	193 (39.5)	295 (40.7)	0.536
Cardiac	171 (16.4)	86 (17.6)	108 (14.9)	0.432
Sepsis	169 (16.2)	91 (18.6)	127 (17.5)	0.489
Surgical	311 (29.9)	132 (27.0)	213 (29.4)	0.51
Trauma	131 (12.6)	45 (9.2)	76 (10.5)	0.114
Respiratory	177 (17.0)	91 (18.6)	111 (15.3)	0.305
Enteral Calories	1457.64 (545.94)	1098.65 (632.99)	1314.54 (748.29)	<0.001
Parenteral Calories	317.51 (578.83)	262.91 (453.08)	444.12 (646.79)	<0.001
Total Calories	1780.17 (444.29)	1369.46 (679.59)	1758.31 (787.96)	<0.001
Calculated energy expenditure	1843.56 (347.63)	2420.13 (604.00)	1958.86 (396.40)	<0.001
Administered calories to energy expenditure	0.98 (0.25)	0.58 (0.28)	0.92 (0.44)	<0.001
Significant parenteral nutrition	127 (12.2)	50 (10.2)	134 (18.5)	<0.001
Total protein delivered	1193.92 (939.76)	561.80 (600.06)	1199.33 (981.55)	<0.001
Daily protein delivered	71.18 (16.79)	45.76 (13.76)	64.69 (17.09)	<0.001
Daily protein, g/kg	0.98 (0.22)	0.48 (0.10)	0.84 (0.22)	<0.001
Daily protein, g/kg, first 3 days	0.93 (0.19)	0.38 (0.19)	0.39 (0.20)	<0.001
Daily protein, g/kg, other days	1.01 (0.24)	0.48 (0.12)	0.93 (0.23)	<0.001

For continuous variables data presented as mean (sd) and discrete variables as *n* (%).

## Supplementary Materials

The following are available online at http://www.mdpi.com/2072-6643/11/1/106/s1. Table S1: Cox regression analysis.

## Abbreviations

ICU	intensive care unit
SOFA	sequential organ failure assessment
REE	resting energy expenditure
