# Supplementary material for: Early Administration of Protein in Critically Ill Patients: A Retrospective Cohort Study
Source: Nutrients. 2019 Jan 7;11(1):106. doi: 10.3390/nu11010106 (PMC6356518; doi:10.3390/nu11010106)
Supplement: Supplementary file 1 [file nutrients-11-00106-s001.pdf]

Table S1- Cox regression analysis

Characteristic	Odds ratio	95% CI	p-value
Age	1.02	1.01 - 1.02	<0.001
Male gender	1.06	0.92 - 1.22	0.394
SOFA score	1.12	1.1 - 1.14	<0.001
Vasopressors	1.06	0.92 - 1.21	0.433
Length of stay	0.97	0.96 - 0.98	<0.001
Parenteral nutrition	1.74	1.46 - 2.07	<0.001
Administered Calories	0.99	0.99-1.00	0.16
Weight	0.99	0.99 - 1.00	0.008
Protein after 3 days	0.93	0.66 - 1.32	0.697
Late low protein	1.24	0.97 - 1.57	0.08
Late High protein	1.21	1.03 - 1.42	0.018
